# The effectiveness of online Eye Movement Desensitization and Reprocessing 2.0 Group Protocol on post-traumatic stress disorders symptoms, depression, anxiety, and stress in individuals who have experienced a traffic accident: a randomized-controlled study

**DOI:** 10.3389/fpsyt.2025.1452206

**Published:** 2025-05-09

**Authors:** Alişan Burak Yasar, İbrahim Gundogmus, Derin Kubilay, Görkem Alban Tunca, Ersin Uygun, Zeynep Zat Çiftçi, Önder Kavakcı

**Affiliations:** ^1^ Department of Clinical Psychology, İstanbul Gelişim University, Istanbul, Türkiye; ^2^ Department of Psychiatry, Ankara Etlik City Hospital, Ankara, Türkiye; ^3^ Private Practice, Istanbul, Türkiye; ^4^ Private Practice, Tekirdag, Türkiye; ^5^ Department of Psychology, İstanbul Bilgi University, Istanbul, Türkiye; ^6^ Institute for Change, Institute for Behavioral Studies, Londra, United Kingdom

**Keywords:** anxiety, depression, EMDR, EMDR 2.0, EMDR 2.0 Group Protocol, online EMDR, online EMDR 2.0, stress

## Abstract

**Introduction:**

EMDR 2.0, an innovative approach rooted in the conventional Eye Movement Desensitization and Reprocessing (EMDR), has garnered attention due to its promising outcomes. The application of EMDR, whether it is EMDR or EMDR 2.0 protocol, in a group format, especially for conditions like Post-Traumatic Stress Disorder, will provide significant opportunities in terms of economic feasibility and accessibility, ultimately leading to widespread use. Building on the established effectiveness of EMDR 2.0 in individual applications, this study examines its impact in group settings. This protocol is designed to provide a structured framework for implementing EMDR 2.0 within group contexts, paving the way for a nuanced understanding of its potential benefits in collective therapeutic settings. This study aims to investigate the efficacy of the online EMDR 2.0 Group Protocol(EMDR 2.0 GP) versus Improving Mental Health Training for Primary Care Residents(mhGAP) on individuals with a history of traffic accidents in a controlled way.

**Methods:**

In this randomized-controlled study sample includes volunteers who were involved in traffic accidents and were given the randomized online EMDR 2.0 GP and mhGAP Stress management module. The participants were given a sociodemographic data form, Depression Anxiety Stress 21 scale (DASS-21) and Impact of Event Scale-Revised (IES-R). Participants were evaluated with measurements before, after and “one month after the application.

**Results:**

The mean age of the participants was 34.80(8.10) years and 88.0% (n=22) were female. The change in DASS-21 Anxiety (h^2^=0.136), Stress (h^2^=0.140), IES-R Avoidance (h^2^=0.134), Hyperarousal (h^2^=0.0148), Total (h^2^=0.223) scores of online EMDR 2.0 GP was determined to be statistically significant compared to the mhGAP group. However, no statistically significant difference was observed in DASS-21 Depression (h^2^=0.017), IES-R Intrusion(h^2^=0.094), scores between the two groups.

**Discussion:**

The RCT of online EMDR 2.0 GP indicated that this newly developed protocol, when applied to groups, may be effective in reducing anxiety, stress, and traumatic symptoms among a non-clinical sample.

**Clinical trial registration:**

https://clinicaltrials.gov/study/, identifier NCT05596903.

## 1 Introduction

EMDR (Eye Movement Desensitization and Reprocessing Therapy) is an evidence-based psychotherapy developed by Francine Shapiro in 1987 for the treatment of trauma and associated disorders (1). Beyond PTSD (2–4), there are many studies that suggest that EMDR can be effective in various psychopathologies such as anxiety disorders (5), major depressive disorder (6), eating disorders (7, 8), and psychotic disorders (9).

Although the positive effects of the standard EMDR therapy protocol have been demonstrated in various studies, a new protocol has been developed to address its limitations and enhance its efficacy across different client groups. (10). This protocol is named EMDR 2.0, and it uses the working memory theory to maximize effectiveness. It was developed to increase the effectiveness, enhance efficiency, and shorten the duration of standard EMDR therapy (10). EMDR 2.0 is basically based on the EMDR standard protocol, but with some differences and add-ons related to the application of EMDR therapy (11).

According to the theory of EMDR 2.0, adequately motivated clients can activate their memory better, so that the working memory taxation is strong enough to decrease the distress induced by aversive memory (10, 12). This approach has 3 components: Motivation, activation, and desensitization. The motivation, which is the first component, is based on the premise that client should receive clear guidelines on actions to be taken for a successful treatment. In the second stage of activation, some sort of triggers can be used to activate memory such as visual, auditory, kinesthetic, olfactory, or gustatory. The aim of this method is to bring the disturbing material into the consciousness by encouraging the client’s participation in the process through triggering objects or experiences (10, 12). When memory is activated in this way, the therapist can use many different strategies to increase the working memory taxation such as doing arithmetic operations while performing rapid eye movements, or keeping a given rhythm with his hands while tapping his feet on the floor at the same time. The distraction based the working memory taxation decreases the vividness and emotionality of the disturbing memory if this material is properly installed to the working memory (12–14). At this stage, clients should focus intently on the different aspects of the memory, while also making an effort to perform distracting tasks (11).

Dual tasking involves simultaneously recalling a distressing memory while performing a second task that taxes working memory (WM). This approach, central to EMDR therapy, creates competition for the brain’s limited WM resources, reducing the emotional intensity and vividness of the memory (15). EMDR 2.0 employs various working memory taxation tasks to enhance working memory reprocessing efficiency (10, 13, 14). These tasks may include language-related exercises, such as counting numbers, or physical tasks. The significance of introducing surprising and unexpected tasks during this stage has also been emphasized. These additional tasks have been shown to increase the level of desensitization by increasing the taxation and activation of working memory, and also prevent the reconsolidation of traumatic memories by using intervention techniques that are not expected by the client (11). EMDR 2.0 approach assumes that if the therapist can successfully make working memory taxation with a dual task while a motivated client keeps the disturbing memory in the working memory, then both of the emotional intensity and the disturbance level of the related memory will drop quickly (11). In dual tasking,memories become less emotional, less vividly aversive, and more strongly reconsolidated in long-term memory (12).

Although the literature investigating the effectiveness of EMDR 2.0 is limited, the results are promising. According to Matthijssen et al. (10), the efficacy of EMDR 2.0 is compared to standard EMDR to measure emotionality and vividness of the disturbing autobiographical memories (10). As a result of this, participants in the EMDR 2.0 group needed fewer session time and a smaller number of sets to reach positive outcomes. In other words, EMDR 2.0 requires less time to achieve a similar effect in desensitizing aversive memories. Beyond individual therapies, group therapies have been established due to the clear benefits such as saving time, economic reasons, and collective healing. To show these significant outcomes, E. Shapiro developed a group practice called the Group Trauma Episode Protocol (G-TEP) based on the EMDR mechanism of action (16). Over time, group applications of G-TEP were found to be effective and frequently usable (17). Afterwards EMDR Group Flash Technique application was conducted (18, 19). EMDR Flash Technique is a therapeutic intervention designed to reduce distress associated with traumatic memories by minimizing the need for clients to directly engage with those memories. In this technique, participants are guided to briefly recall a positive memory or mental image while simultaneously maintaining minimal awareness of the distressing memory. This method aims to facilitate the desensitization of traumatic experiences through rapid bilateral stimulation (e.g., eye movements or blinks), helping to lower emotional intensity without requiring extensive exposure to the traumatic content (18).

This study has demonstrated that EMDR 2.0 GP can be easily applied to a larger number of participants, and, with fewer therapists, it effectively alleviates traumatic symptoms in a much shorter time than traditional structured long group therapies (20) Moreover, this has been achieved even in groups where managing difficult topics like traumatic stress and handling group dynamics delicately is required. There are some reasons that lead to the use of EMDR 2.0 compared to EMDR. First of all, it is important that the group application of EMDR 2.0 is very new and its effectiveness is shown. In addition, comfortable and safe use provides advantages for group application. In addition, having a group protocol for online use was also an important advantage. In this context, it is important to demonstrate the effectiveness of group application of EMDR 2.0.

In light of the positive contributions of the EMDR Group Flash Technique Protocol, the online EMDR 2.0 Pilot Study for Group Protocol was conducted (21). This study involved seven individuals with a prior traffic accident history, and the results of the pilot study demonstrated a significant reduction in depression and stress-related symptoms of the participants. Additionally, there was a statistically significant decrease in the symptoms of post-traumatic stress disorder that are re-experiencing, avoidance and a decrease in hyperarousal symptoms was also observed in the participants.

In this study, the online EMDR 2.0 GP protocol developed by Yaşar and colleagues was employed (21). This study aims to investigate the efficacy of the online EMDR 2.0 Group protocol on individuals with a history of traffic accidents in a controlled way. The first hypothesis is that the online EMDR 2.0 Group Protocol will decrease symptoms of depression, anxiety, and stress. The second hypothesis posits a significant reduction in symptoms of re-experiencing, avoidance, and hyperarousal. Finally, it hypothesizes that online EMDR 2.0 will lead to greater reductions in these symptoms compared to the Mental Health Gap Action Programme (mhGAP).

## 2 Material and methods

### 2.1 Sample

**Figure 1:**
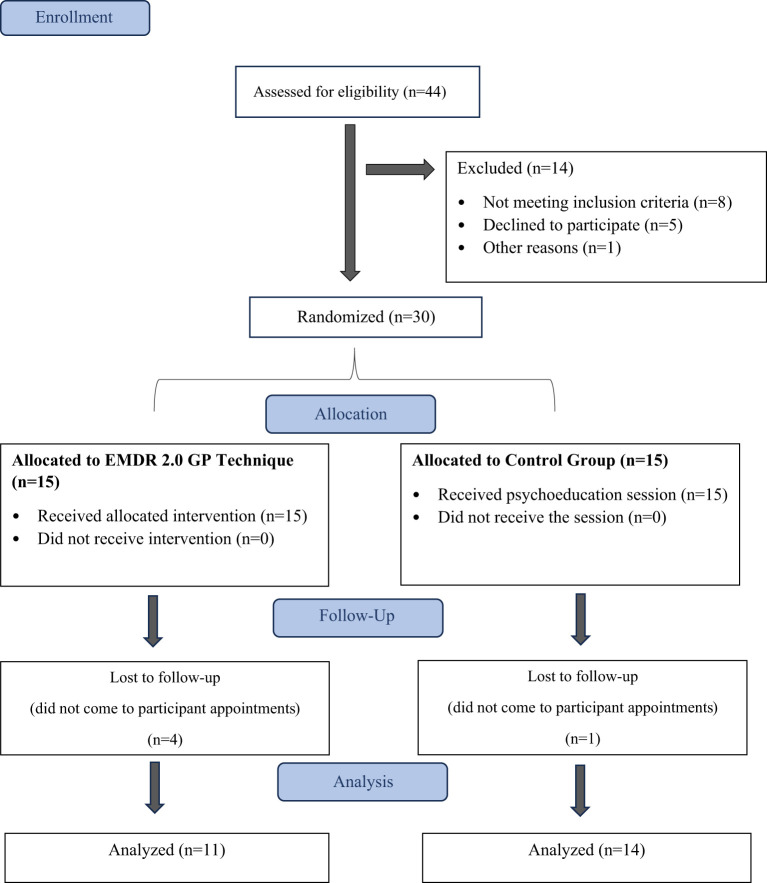
Flowchart of participants through each stage of the study.

In this randomized controlled trial, we assessed the effectiveness of the online EMDR 2.0 GP and mhGAP through pre- and post-intervention evaluations, with a subsequent follow-up conducted one month after the interventions were administered. We initiated the study by extending online invitations to participants who had experienced traumatic symptoms stemming from traffic accidents. Out of the 44 eligible participants, 35 expressed volunteers in participating in the study. However, 5 individuals were unable to take part due to medical or technical constraints. Consequently, the study resulted in a final group of 30 volunteers who met the predefined inclusion criteria (**Figure 1**).

The inclusion criteria were as follows: (a) being over 18 years of age, (b) having experienced a traffic accident between 6 months and 10 years ago, (c) not having a mental disability resulting from a traffic accident, (d) the absence of a psychotic disorder such as schizophrenia or bipolar disorder, (e) not having experienced severe head trauma, (f) having the necessary technological proficiency and equipment to participate in the study, and (g) volunteering to take part in the research.

We carefully reviewed the CONSORT statement and its associated checklist to ensure all components of the study are adequately explained. The checklist was used as a guide to verify that key methodological elements, including participant recruitment, randomization process, intervention details, outcome measures, and statistical analysis, were thoroughly described. This review helped identify and clarify areas requiring additional detail, such as informed consent procedures and the differentiation between the intervention and control groups. By adhering to the checklist, we aimed to provide a clear and comprehensive presentation of the study design and execution, ensuring transparency and replicability.

This research was approved by the Istanbul Gelisim University Ethics Committee (03.06.2022/2022-10). All stages of the study were carried out in accordance with the Declaration of Helsinki. The protocol was registered at the National Library of Medicine Trial Registry (NCT05596903). All participants provided informed consent before participating in the study. Informed consent was obtained for both the online EMDR 2.0 GP and mhGAP conditions, ensuring that participants were aware of the procedures, potential risks, and benefits of the study.

### 2.2 Study design

The study, coordinated by the Academy of Therapeutic Science, was carried out over three consecutive days with total of 3 sessions in October 2022. Individuals minimally attended 2 sessions. To recruit individuals who had experienced car accidents, announcements were posted on social media platforms, outlining the research objectives. Volunteers provided their contact information through a Google Form. They were then interviewed, informed about the study, and assessed for eligibility. Eligible participants completed a Google Form with data collection tools. These participants were randomly assigned to groups for the online EMDR 2.0 GP and mhGAP interventions, administered online. Those unable to complete the interventions were excluded. Participants were requested to complete follow-up forms one week and one-month post-intervention. Individuals who were unable to complete the interventions at this stage were excluded from the study. The collected data was processed and subjected to statistical analysis.

### 2.3 Procedure

#### 2.3.1 The mhGAP Stress management module

The mhGAP program, also known as “Improving Mental Health Training for Primary Care Residents in Turkey,” is an initiative by the World Health Organization (WHO) aimed at enhancing mental health services within primary healthcare settings. This program encompasses stress management and its related disorders as a vital component. In Turkey, the mhGAP training program for primary care family physicians is an ongoing collaborative effort between the Ministry of Health and the WHO Turkey office. One of the primary strategies advocated in the management of stress and its associated disorders within this program is termed “Psychoeducation for stress and associated disorders.” The main goal of this training is to facilitate an understanding of the origins of an individual’s stress symptoms and to provide insight into the cluster of symptoms that arise in response to stressful events. Consequently, this approach helps alleviate the tension brought about by the individual’s symptoms. In the context of our study, one of our researchers, who also serves as a trainer within this module, administered the recommended psychoeducation sessions on stress-related disorders to the control group.

#### 2.3.2 Online EMDR 2.0 GP

In this study, the online EMDR 2.0 GP protocol developed by Yaşar and colleagues was implemented (21). The duration of the first session was 90 minutes, while each of the subsequent two sessions lasted for 60 minutes. Each session had one therapist and two co-therapists in attendance. Co-therapists were prepared to intervene if there was any need for dissociation or individual interventions. An overview of the online EMDR 2.0 GP protocol is provided below:

##### 2.3.2.1 Introduction and consent

After a brief introduction, participants were informed about the protocol. Informed consent was obtained from participants for registration. A visual bilateral stimulus screen, displaying a point moving horizontally to the right and left, which would be used in the application, was introduced. Traumatic memories were discussed, and a Safe Place exercise was conducted to be used in case of any dissociation during the sessions.

Participants were asked to identify three distressing images related to the traffic accident. During this stage, participants were encouraged to visualize their memories but were not required to narrate them to the therapist or other participants. As a preventive measure against intra-group retraumatization, and as an opportunity offered by online EMDR 2.0 GP, participants were able to complete the therapy process without sharing the details of their traumatic memories. In group sessions, no participant was exposed to the traumatic experiences of others. Participants were requested to assign Subjective Units of Disturbance (SUD) scores out of 10 to each of the identified images. The study adhered to the CONSORT guidelines to ensure transparency and reproducibility.

##### 2.3.2.2 Online EMDR 2.0 GP application

Explanation and Application of Guidelines:

- Participants focused on one image in each set and followed the horizontally moving point on the screen for bilateral stimulation.
- Dual tasks were applied during bilateral stimulation to activate working memory taxation. The task changed approximately every 3 sets, with no strict rule for task-switching timing.
- Participants were instructed to concentrate on both the task and the image during bilateral stimulation.

##### 2.3.2.3 Performance assessments

After every two sets, participants provided:

- A rating for their Focusing on the Memory (FM) score out of 100, indicating how long they could stay focused on the memory during the bilateral stimulation in the last two sets.
- A score for their Performing the Task (PT) out of 100, indicating how well they could focus on the task during the bilateral stimulation in the last two sets.
- The SUD score for the selected image.
- Participants were instructed to record these scores (SUD, FM, and PT) on their participant forms.

In this stage, FM (Focusing on the Memory) and PT (Performing the Task) scores were applied to measure the effectiveness of online EMDR 2.0 according to its theory. The goal was for participants to perform both assessments as effectively as possible. Participants who showed low scores below 50 during breaks due to minimal focus on the memory or not completing the task were motivated, and the importance of this was briefly reminded. This was done to ensure that everyone applied online EMDR 2.0 efficiently and appropriately in this group session.

##### 2.3.2.4 Progressive image work

Participants continued to work on the images until a progressive reduction in distress, as measured by the SUD scores, was achieved.

Participants who reached a SUD score of 0 out of 10 for the first image proceeded to the next one, while others continued to work on the first image.

##### 2.3.2.5 Review of SUD scores

At the end of the application, participants were asked to review all their SUD scores from the first set to the last one. A brief evaluation was conducted regarding relaxation during the memories.

Completion and Stabilization:

The application concluded with stabilization and orientation exercises.

##### 2.3.2.6 Session progression

The number of sets performed varied depending on the available time during each session. In this study, an average of 7 sets was performed on the first day, 8 sets on the second day, and 6 sets on the last day.

##### 2.3.2.7 Distracting instructions

Throughout the study, distracting instructions were given, such as listening to various songs, counting repetitive words in songs, performing bodily movements, and counting backward the letters of specific words. These tasks, for example, included playing well-known upbeat songs in Turkey such as “Senden Başka” and “Salla.” In another task, participants were asked to count how many times the words “başka” or “salla” appeared in these songs. In a different task, they were instructed to spell several words backward syllable by syllable. In another task, participants were required to perform specific hand and arm movements rhythmically. These tasks were divided into 5 stages, and during each session, the entire group progressed through them one by one simultaneously. The tasks were progressively made more challenging throughout the sessions.

##### 2.3.2.8 Dual task integration

In the last two days, participants were asked to perform dual tasks simultaneously during certain sets. Interventions between sets were carried out based on changes in FM and PT scores.

##### 2.3.2.9 Post-session evaluation

After each session, participants received an evaluation form, where they reported their Subjective Units of Disturbance (SUD), Focusing on the Memory (FM) scores, and Performing the Task (PT) scores for the images they worked on. Additionally, participants were asked if they had transitioned to other memories, and if so, relevant scores were recorded.

### 2.4 Data collection tools

#### 2.4.1 Sociodemographic form

This semi-structured data collection form is administered by the researchers to gather demographic information from the participants. It includes details such as age, gender, and data related to their experience of trauma resulting from traffic accidents, in accordance with the relevant literature.

#### 2.4.2 Impact of event scale (IES-R)

Developed by Weis and Marmara in 1997, this scale is used to measure the psychological impact of events on individuals participating in the study. (22). It is a self-report instrument with a five-point Likert-type response scale and consists of 22 items. The scale assesses the extent of exposure to events across three domains: “Intrusion,” “Avoidance,” and “Hyperarousal.” Turkish validity and reliability studies for this scale have been conducted, as documented by 23 (23).

#### 2.4.3 Depression-anxiety-stress scale-21

This scale is employed to evaluate symptoms of depression, anxiety, and stress among the study participants. It comprises 21 items, each assessed on a 4-point Likert-type scale. Scores for each subdimension can range from 0 to 21. The Turkish validity and reliability of this scale were established by Saricam in 2018 (24).

### 2.5 Statistical analysis

In order to obtain a clinically and statistically significant difference, it was decided to include a total of 20 patients (10 controls and 10 applications) with a significance level of 5%, power of 80% and an effect size of 0.5.

The statistical analysis for this study was conducted using the SPSS 22 software. Descriptive data were presented using frequency and percentage for categorical variables. Mean and standard deviation were used for continuous variables. The Pearson Chi-square test was utilized to compare categorical variables. For continuous variables that met parametric assumptions, Student’s t-test was applied. For continuous variables that did not meet parametric assumptions, the Mann-Whitney U test was used. Repeated measures analysis of variance (ANOVA) was employed to determine whether the online EMDR 2.0 GP and mhGAP procedures, when administered separately, resulted in significant changes in the dependent variables. This analysis also assessed whether there were differences in the changes between the two groups. The Mauchly test was used to check whether the sphericity assumption had been violated. When the Mauchly test indicated a violation of the sphericity assumption, the Greenhouse-Geisser adjustment was applied, and the corrected results were reported. Effect sizes were determined using η² (eta-squared) and Cohen’s d. These measures help assess the practical significance of observed differences. A p-value of <.05 was considered statistically significant.

## 3 Results

### 3.1 Participant characteristics

The participants in the study had a mean age of 34.80 (8.10) years, with 88.0 percent (n=22) being female. **Table 1** compares sociodemographic and traffic accident-related ratings between the pre-intervention online EMDR 2.0 GP and mhGAP groups. As a result, there was no significant difference between the two groups when sociodemographic and traffic accident-related ratings were compared (p>.05).

**Table 1:** Baseline characteristics of mhGAP and Group EMDR 2.0 participants.

	mhGAP (n=11)	Group EMDR 2.0 (n=14)	p value
**Age; year, Mean (SD)**	34.63 (5.63)	33.64 (9.73)	0.474
**Gender (Female); n (%)**	10 (90.9%)	12 (85.7%)	0.692
**Marital Status (Single); n (%)**	4 (36.4%)	5 (35.7%)	0.660
**Employment (Yes); n (%)**	10 (90.9%)	10 (71.4%)	0.227
**Education (University); n (%)**	10 (90.9%)	13 (92.9%)	0.859
**Time after the accident; month, Mean (SD)**	31.72 (33.20)	38.71 (37.81)	0.633
**Smoking (Yes); n (%)**	1 (9.1%)	5 (35.7%)	0.122
**Alcohol (Yes); n (%)**	5 (45.5%)	6 (42.9%)	0.897
**Substance (Yes); n (%)**	1 (9.1%)	0	0.250

mhGAP, Mental Health Gap Action Program; SD, Standard deviation.

Bold text is statistically significant.

### 3.2 Pre-intervention comparisons

There was no statistical difference between the two study groups pre-intervention in DASS-21 Anxiety (p=.977), Depression (p=.967), Stress (p=.565) subscale scores, IES-R Intrusion (p=.667), Avoidance (p=.885), Hyperarousal (p=.330), and total (p=.586) scores.

### 3.3 Between-group comparisons

**Table 2** compares DASS-21 Anxiety, Depression, and Stress subscale scores and Impact of Event Scale-Revised Intrusion, Avoidance, Hyperarousal, and total scores in the online EMDR 2.0 and mhGAP groups before, one week, and one month after the study, within and between groups. As a result, there was a statistically significant difference in the DASS-21 Depression (F_(2-20)_=7.567, p=.005, η²=0.431), Anxiety (F_(2-20)_=6.799, p=.015, η²=0.405), IES-R Intrusion (F_(2-20)_=11.929, p=.001, η²=0.544), Avoidance (F_(2-20)_=6.853, p=.009, η²=0.457), Hyperarousal (F_(2-20)_=6.661, p=.007, η²=0.400), Total (F_(2-20)_=14.550, p<.001, η²=0.593) subscale scores in the mhGAP group, while no statistically significant difference was found in the DASS-21 Stress (F_(2-20)_=2.985, p=.075, η²=0.230) scores.

**Table 2:** Descriptive statistics for outcome variables in the mhGAP and Group EMDR 2.0.

	Methods	Pre-measurement	Post-measurement	Follow-up Measurement	Between Time p value and Effect Size (η²)	Between Groups p value and Effect Size by the time (η²)
DASS-21; Mean (SD)						
**Anxiety**	mhGAP	6.18 (3.12)	4.81 (3.81)	4.36 (2.83)	**F2-20 = 6.799** **P=0.015, η²=0.405**	**F2-46 = 3.628** **P=0.037** η²=0.136
	Group EMDR 2.0	6.14 (3.57)	2.71 (2.52)	2.42 (2.82)	**F2-26 = 21.096** **P<0.001, η²=0.619**	
**Depression**	mhGAP	8.27 (2.86)	4.72 (4.38)	5.72 (2.68)	**F2-20 = 7.567** **P=0.005, η²=0.431**	**F2-46 = 0.396** p=0.674 η²=0.017
	Group EMDR 2.0	8.21 (3.80)	3.57 (2.68)	4.50 (3.39)	**F2-26 = 10.445** **P=0.001, η²=0.446**	
**Stress**	mhGAP	8.54 (1.80)	7.54 (3.14)	6.45 (2.87)	**F2-20 = 2.985** **P=0.075, η²=0.230**	**F2-46 = 3.737** **P=0.035** η²=0.140
	Group EMDR 2.0	9.14 (2.98)	5.14 (3.63)	4.42 (2.97)	**F2-26 = 19.277** **P<0.001, η²=0.597**	
Impact of Events Scale-Revised; Mean (SD)						
**Intrusion**	mhGAP	11.90 (5.50)	7.45 (4.61)	5.36 (4.34)	**F2-20 = 11.929** **P=0.001, η²=0.544**	**F2-46 = 2.382** **P=0.115** η²=0.094
	Group EMDR 2.0	12.92 (6.01)	4.42 (4.14)	3.42 (3.03)	**F2-26 = 31.489** **P<0.001, η²=0.708**	
**Avoidance**	mhGAP	12.54 (4.48)	9.27 (3.55)	9.18 (4.35)	**F2-20 = 6.853** **P=0.009, η²=0.457**	**F2-46 = 3.550** **P=0.042** η²=0.134
	Group EMDR 2.0	12.28 (4.33)	6.28 (3.49)	4.57 (3.56)	**F2-26 = 22.294** **P<0.001, η²=0.632**	
**Hyperarousal**	mhGAP	10.09 (4.92)	7.27 (4.73)	6.63 (5.44)	**F2-20 = 6.661** **P=0.007, η²=0.400**	**F2-46 = 3.998** **P=0.025** η²=0.148
	Group EMDR 2.0	11.85 (3.95)	5.85 (5.26)	4.85 (3.77)	**F2-26 = 33.021** **P<0.001, η²=0.718**	
**Total**	mhGAP	34.54 (13.58)	24.00 (11.13)	21.18 (12.75)	**F2-20 = 14.550** **P<0.001, η²=0.593**	**F2-46 = 6.5594** **P=0.003** η²=0.223
	Group EMDR 2.0	37.00 (8.80)	16.57 (9.99)	12.42 (8.81)	**F2-26 = 73.111** **P<0.001, η²=0.849**	

mhGAP: Mental Health Gap Action Program, SD: Standard deviation, DASS-21: Depression Anxiety Stress Scale-21, *:p<0.05.

Bold text is statistically significant.

There was a statistically significant difference in the DASS-21 Anxiety (F_(2-26)_=21.096, p<.001, η²=0.619), Depression (F_(2-26)_=10.445, p<.001, η²=0.446), Stress (F_(2-26)_=19.277, p<.001, η²=0.597), IES-R Intrusion (F_(2-26)_=31.489, p<.001, η²=0.708), Avoidance (F_(2-26)_=22.294, p<.001, η²=0.632), Hyperarousal (F_(2-26)_=33.021, p<.001, η²=0.718), Total (F_(2-26)_=14.550, p<.001, η²=0.593) subscale scores in the online EMDR 2.0 GP.

### 3.4 Time and group interactions

**Figure 2:**
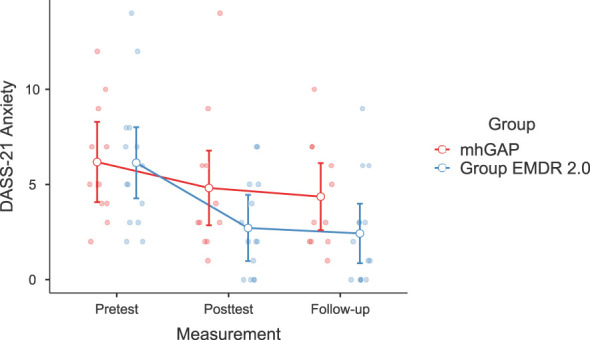
Graph of change of DASS-21 Anxiety score between two applications.

**Figure 3:**
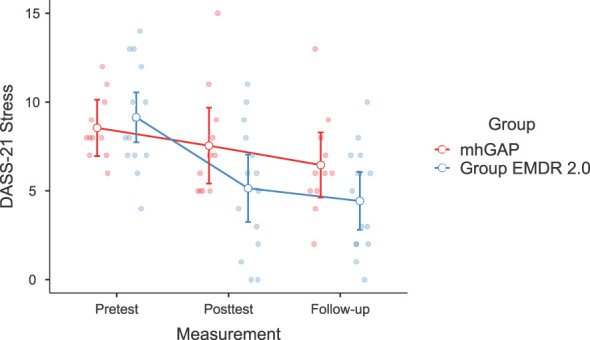
Graph of change of DASS-21 Stress score between two applications.

**Figure 4:**
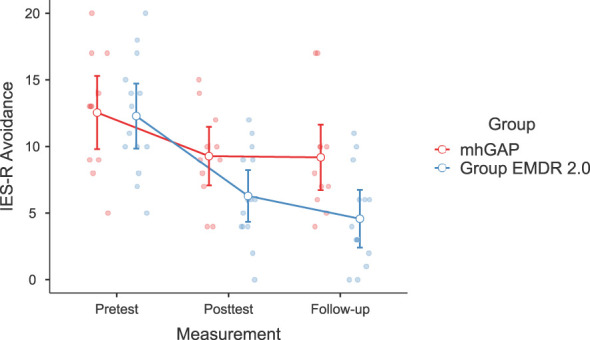
Graph of change of IES-R Avoidance score between two applications.

**Figure 5:**
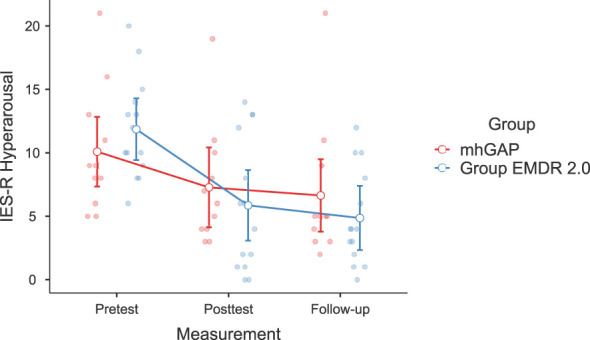
Graph of change of IES-R Hyperarousal score between two applications.

**Figure 6:**
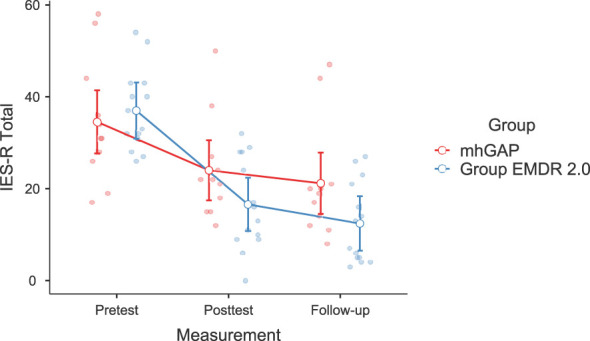
Graph of change of IES-R Total score between two applications.

**Figure 7:**
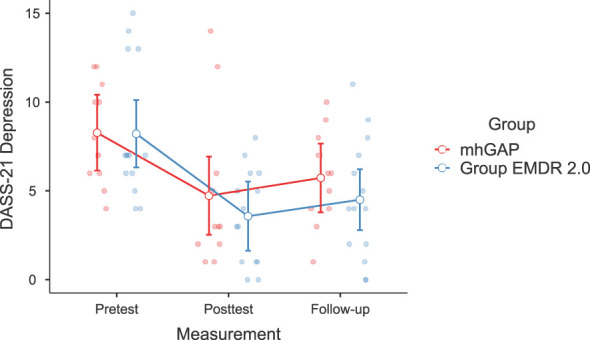
Graph of change of DASS-21 Depression score between two applications.

**Figure 8:**
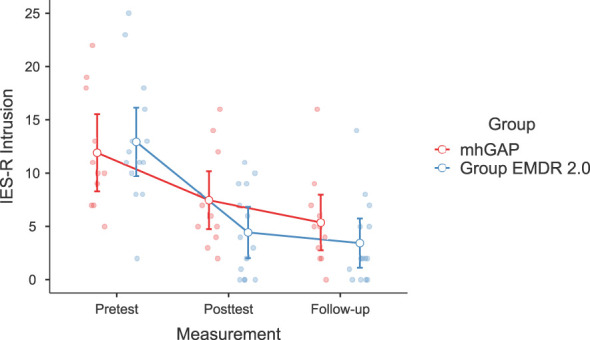
Graph of change of IES-R Intrusion score between two applications.

Significant changes were noted in DASS-21 Anxiety (F(2-46)=3.628, p=.037, η²=0.136, **Figure 2**) and Stress (F(2-46)=3.737, p=.035, η²=0.140) **Figure 3**), IES-R Avoidance (F(2-46)=3.550, p=.042, η²=0.134, **Figure 4**), Hyperarousal (F(2-46)=3.998, p=.025, η²=0.0148, **Figure 5**), Total (F(2-46)=6.594, p=.003, η²=0.223, **Figure 6**) scores of the online EMDR 2.0 GP were determined to be statistically significantly different compared to the mhGAP group. However, no statistically significant difference was observed in the DASS-21 Depression (F(2-46)=0.396, p=.674, η²=0.017, **Figure 7**), IES-R Intrusion (F(2-46)=2.382, p=.115, η²=0.094, **Figure 8**), scores between the two groups.

## 4 Discussion

In the present randomized controlled trial, the hypothesis that the online EMDR 2.0 GP would reduce depression, re-experiencing, avoidance, hyperarousal and stress symptoms more than mhGAP was investigated in individuals who had previously experienced a traffic accident. According to the result of the study, online EMDR 2.0 may reduce symptoms of traumatic stress, anxiety, and depression. These effects are assumed to be related to increased working memory taxation, which is a core mechanism of the EMDR 2.0 protocol. Furthermore, in the group therapy sessions conducted for trauma, participants appeared to benefit from the intervention with minimal disclosure of their traumatic experiences and without observable signs of mutual retraumatization. The current results have clearly demonstrated that the online EMDR 2.0 GP application effectively alleviates symptoms in individuals emotionally impacted by traffic accidents.

A statistically significant reduction was observed in DASS-21 Anxiety, Depression, and Stress scores, supporting the first hypothesis. This decrease remained consistent in follow-up assessments conducted one week and one month after the intervention, indicating a sustained therapeutic effect of the online EMDR 2.0 GP. According to the first hypothesis, a statistically significant reduction was observed in the DASS-21 Anxiety, Depression, and Stress. In their pilot study, they showed that individuals who had encountered traffic accidents exhibited reduced symptoms of depression following the application of online EMDR 2.0 GP (21). Numerous articles have provided evidence for the effectiveness of a single EMDR session (6, 25–27) or group settings (28–30) in diminishing depression. However, this study also introduces online EMDR 2.0 GP as an alternative approach for reducing depression symptoms.

The second hypothesis was supported, with a statistically significant reduction observed in the Impact of Event Scale-Revised subscales of intrusion, avoidance, and hyperarousal in the online EMDR 2.0 group. This reduction remained consistent in both the one-week and one-month follow-up assessments compared to baseline scores. In their preliminary investigation, Yaşar and colleagues demonstrated that the application of online EMDR 2.0 GP led to an anticipated reduction in the re-experiencing, avoidance, and hyperarousal symptoms associated with traumatic memories among the participants (21). This reduction was observed when comparing these symptoms to their initial assessment before treatment. Several articles suggest that EMDR is an effective method for diminishing anxiety-related symptoms of participants (31–35) due to the strength of processing past anxiety-evoking memories. These findings suggest that online EMDR 2.0 GP, being a quicker and effective approach, also leads to a reduction in these symptoms.

For the final hypothesis, online EMDR 2.0 was expected to result in greater reductions in anxiety and stress symptoms compared to mhGAP. The results supported this expectation, as participants in the EMDR 2.0 condition reported significantly greater improvements in anxiety and stress. However, no statistically significant difference was found between the two interventions in terms of depressive symptoms. Individuals who received online EMDR 2.0 GP treatment exhibited lower levels of anxiety and stress-related symptoms when compared to those in the mhGAP group. The final hypothesis proposed the notion that EMDR 2.0 GP represents a more effective approach compared to mhGAP in reducing symptoms associated with trauma, such as stress, anxiety, and depression. mhGAP aims to address the significant treatment gap that exists in mental health care by providing guidance and resources for the integration of mental health services into primary healthcare systems (36). In this study, it was used as a psychoeducation tool for raising awareness of the participants on traumatic symptoms (36). Also, it was used as a psychoeducation tool to raise participants’ awareness of traumatic symptoms. In a study, Yaşar and his colleagues sought to assess the effectiveness of the EMDR Flash technique by comparing it with mhGAP (19). Their findings indicated that when administered to individuals who had experienced traffic accidents, the EMDR Flash technique proved successful in enhancing symptoms related to anxiety, intrusion, avoidance, overall traumatic stress, and mental quality of life for a minimum of one month. The decline in psychometric measurements among the participants, in comparison to the baseline assessment phase, persisted across measurements conducted at 1 week and 1-month post-intervention. Similarly, the effectiveness of online EMDR 2.0 GP observed in this study is consistent with the current findings supporting psychoeducational approaches in trauma treatment.

Another important issue in this study is that the EMDR 2.0 GP application, which is a group psychotherapy application, can also be applied online. This approach offers significant advantages, such as expanding client reach by eliminating geographical barriers and reducing costs and time investment. In addition, individuals participating in therapy in the comfort of their own homes can make the process more accessible, especially for people with disorders such as anxiety or social phobia. However, it is difficult to predict whether the online application affects the effectiveness of the EMDR 2.0 GP application. Nevertheless, there are also disadvantages such as the therapist not being able to fully observe body language and facial expressions, the risk of interruption of the session due to internet connection or technical problems. In addition, the inability to intervene quickly with the client in crisis situations such as dissociation and the possibility of the therapeutic alliance developing weaker than in face-to-face therapies can be counted among the limitations.

Beyond its application for PTSD, EMDR 2.0 could offer a valuable solution for improving participant engagement and emotional stability, particularly in cases of challenging conditions like dissociative syndromes and depression, where recollection of positive memories is not easily accessible. In contrast to the standard EMDR protocol, individuals undergoing EMDR 2.0 report experiencing notably positive emotions. This can be regarded as one of the strengths of EMDR 2.0, as it offers reduced exposure to traumatic memories and a comparatively pleasant therapeutic experience. The results of the current research need to be interpreted within certain limitations. The primary limitation of the study is its small sample size, which restricts the generalizability of the finding. It is recommended to include more diverse samples in future studies, taking into account factors such as age and gender. In addition, another limitation is that the current sample of individuals who experienced trauma did not include individuals who were clinically diagnosed with PTSD. Future studies may benefit from incorporating clinical samples or investigating other challenging scenarios for a more comprehensive understanding. Additionally, the follow-up period of the participants was extended only for one month after application and no measurements were made afterwards. Future research may benefit from longer follow-up periods and ongoing assessments to capture potential changes or improvements over a longer period of time. Finally, the fact that the measurements were made using self-report methods can be considered as another limitation. It should also be kept in mind that differences in trauma durations may create a limitation. In our pilot study, we have demonstrated the applicability and effectiveness of the online EMDR 2.0 GP application protocol. This study, in addition to previous research on online EMDR 2.0 GP applications (10), which primarily focused on individual interventions, establishes the success of online EMDR 2.0 GP in ameliorating symptoms of depression, anxiety, and stress within a larger sample.

In this study, online EMDR 2.0 GP was applied for a larger group of individuals, and a decrease was observed in the participants’ depression, anxiety levels, stress-related symptoms, avoidance, re-experiencing, and hyperarousal symptoms associated with traumatic memories. These results are promising for the effectiveness of the developed online EMDR 2.0 GP application. Furthermore, the brief exposure to traumatic memories and the absence of a detailed account of the traumatic event make this approach well-suited for group settings. In scenarios with constraints on time and resources, such as natural disaster-stricken areas, where extended therapy sessions are not feasible, online EMDR 2.0 GP presents a substantial advantage. Additionally, the ability to apply online EMDR 2.0 GP online further extends its reach to individuals who may be geographically distant or face other barriers to accessing therapy. To conclude, the present research is the first clinical randomized control study to compare the effectiveness of the online EMDR 2.0 GP application with the mhGAP module, a structured application, on the assessment and management of stress-related conditions. We believe that these results will contribute to the reliability and applicability of the online EMDR 2.0 GP in future.

## Data Availability

The raw data supporting the conclusions of this article will be made available by the authors, without undue reservation.

## References

[1] Shapiro F. Eye movement desensitization and reprocessing (EMDR) therapy: basic principles, protocols, and procedures. New York, Ny: Guilford: Guilford Publications (2017).

[2] Maxfield L Hyer L. The relationship between efficacy and methodology in studies investigating emdr treatment of ptsd. J Of Clin Psychol. (2002) 58:23–41. doi: 10.1002/jclp.v58:1 11748595

[3] Valiente-Gómez A Moreno-Alcázar A Treen D Cedrón C Colom F Perez V. Emdr beyond ptsd: A systematic literature review. Front In Psychol. (2017) 8:1668. doi: 10.3389/fpsyg.2017.01668 PMC5623122 29018388

[4] Seidler GH Wagner FE. Comparing the efficacy of emdr and trauma-focused cognitive-behavioral therapy in the treatment of ptsd: A meta-analytic study. psychol Med. (2006) 36:1515–22. doi: 10.1017/S0033291706007963 16740177

[5] Faretta E Dal Farra M. Efficacy of emdr therapy for anxiety disorders. J Of Emdr Pract And Res. (2019) 13:325–32. doi: 10.1891/1933-3196.13.4.325

[6] Gauhar YWM. The efficacy of emdr in the treatment of depression. J Of Emdr Pract And Res. (2016) 10:59–69. doi: 10.1891/1933-3196.10.2.59

[7] Yaşar AB Abamor AE Usta FD Taycan SE Kaya B. Two cases with avoidant/restrictive food intake disorder (Arfid): effectiveness of emdr and cbt combination on eating disorders (Ed). Klinik Psikiyatri Dergisi. (2019) 22:493–500. doi: 10.5505/kpd.2019.04127

[8] Balbo M Zaccagnino M Cussino M Civilotti C. Eye movement desensitization and reprocessing (Emdr) and eating disorders: A systematic review. Clin Neuropsychiatry. (2017) 14:321–9.

[9] Oztanriover S Yasar AB Gundogmus I Altunbas FD. Effects of eye movement desensitization and reprocessing on disease symptoms and functionality in patients with psychotic disorders/psikotik bozukluk hastalarinda goz hareketleriyle duyarsizlastirma ve yeniden isleme terapisinin hastalik belirtilerine ve hastalarin islevselliklerine etkisi: olgu serisi. Anadolu Psikiyatri Dergisi. (2019) 20:522–30. doi: 10.5455/apd.20832

[10] Matthijssen SJ Brouwers T Van Roozendaal C Vuister T De Jongh A. The effect of emdr versus emdr 2.0 on emotionality and vividness of aversive memories in A non-clinical sample. Eur J Of Psychotraumatol. (2021) 12:1956793. doi: 10.1080/20008198.2021.1956793 34567439 PMC8462855

[11] Matthijssen SJ Lee CW De Roos C Barron IG Jarero I Shapiro E. The current status of emdr therapy, specific target areas, and goals for the future. J Of Emdr Pract And Res. (2020) 14:241–84. doi: 10.1891/EMDR-D-20-00039

[12] Van Den Hout MA Engelhard IM. How does emdr work? J Of Exp Psychopathol. (2012) 3:724–38. doi: 10.1016/j.brat.2012.02.001

[13] Baddeley A. Working memory. Science. (1992) 255:556–9. doi: 10.1126/science.1736359 1736359

[14] Baddeley A. Working memory. . Curr Biol. (2010) 20:R136–40. doi: 10.1016/j.cub.2009.12.014 20178752

[15] Mertens G Lund M Engelhard IM. The effectiveness of dual-task interventions for modulating emotional memories in the laboratory: A meta-analysis. Acta Psychologica. (2021) 220:103424. doi: 10.1016/J.Actpsy.2021.103424 34619553

[16] Shapiro E. The emdr group traumatic episode protocol. New York, Ny: Guilford: Guilford Publications (2013).

[17] Robinson RM Kaptan SK. The future possibilities of group emdr therapy. In: Emdr group therapy: emerging principles and protocols to treat trauma and beyond. Springer Publishing (2023).

[18] Yaşar AB Gündoğmuş I Gündüz A Konuk E. The effects of single session emdr flash technique group application on traumatic symptoms. Israel J Of Psychiatry. (2021) 58:41–6.

[19] Yaşar AB Konuk E Kavakçı Ö. Uygun E Gündoğmuş I Taygar AS. A randomized-controlled trial of emdr flash technique on traumatic symptoms, depression, anxiety, stress, and life of quality with individuals who have experienced A traffic accident. Front In Psychol. (2022) 13:845481. doi: 10.3389/fpsyg.2022.845481 PMC8987710 35401305

[20] Barkowski S Schwartze D Strauss B Burlingame GM Rosendahl J. Efficacy of group psychotherapy for anxiety disorders: A systematic review and meta-analysis. Psychother Res. (2020) 30:965–982.). doi: 10.1080/10503307.2020.1729440 32093586

[21] Yaşar AB Kavakçı Ö. Çiftçi ZZ Tunca GA Uygun E Gündoğmuş I. The effectiveness of online EMDR 2.0 group protocol on posttraumatic stress disorder symptoms, depression, anxiety, and stress in individuals who have experienced a traffic accident: a preliminary study. Journal of EMDR Practice and Research. (2023) 17(3): 171–84.

[22] Weiss DS. The impact of event scale: revised. In: Cross-cultural assessment of psychological trauma and ptsd. Boston, MA: Springer (2007).

[23] Çorapçıoğlu A Yargıç I Geyran P Kocabaşoğlu N Etkisi Olayların Ies-R Ölçeği. Türkçe versiyonunun geçerlilik ve güvenilirliği. In: Yeni symposium (2006) 14–22.

[24] Saricam H. The psychometric properties of turkish version of depression anxiety stress scale-21 (Dass-21) in health control and clinical samples. J Of Cogn Behav Psychother And Res. (2018) 7:19–30. doi: 10.5455/JCBPR.274847

[25] Uribe MER Ramírez EOL Mena IJ. Effect of the emdr psychotherapeutic approach on emotional cognitive processing in patients with depression. Spanish J Of Psychol. (2010) 13:396–405. doi: 10.1017/S1138741600003954 20480706

[26] Moghadam MB Moghadam AB Salehian T. Efficacy of eye movement desensitization and reprocessing (Emdr) on depression in patients with myocardial infarction (Mi) in A 12-month follow up. Iranian J Of Crit Care Nurs. (2015) 7:221–6.

[27] Wood E Ricketts T Parry G. Emdr as A treatment for long-term depression: A feasibility study. Psychol And Psychotherapy: Theory Res And Pract. (2018) 91:63–78. doi: 10.1111/papt.2018.91.issue-1 PMC5836996 28834138

[28] Hase M Balmaceda UM Hase A Lehnung M Tumani V Huchzermeier C. Eye movement desensitization and reprocessing (Emdr) therapy in the treatment of depression: A matched pairs study in an inpatient setting. Brain And Behav. (2015) 5:E00342. doi: 10.1002/brb3.2015.5.issue-6 PMC4467776 26085967

[29] Osorio A Pérez MC Tirado SG Jarero I Givaudan M. Randomized controlled trial on the emdr integrative group treatment protocol for ongoing traumatic stress with adolescents and young adults patients with cancer. Am J Of Appl Psychol. (2018) 7:50–6. doi: 10.11648/j.ajap.20180704.11

[30] Perilli S Giuliani A Pagani M Mazzoni GP Maslovaric G Maccarrone B. Emdr group treatment of children refugees—A field study. J Of Emdr Pract And Res. (2019) 13:143–55. doi: 10.1891/1933-3196.13.2.143

[31] Goldstein AJ De Beurs E Chambless DL Wilson KA. Emdr for panic disorder with agoraphobia: comparison with waiting list and credible attention-placebo control conditions. J Of Consulting And Clin Psychol. (2000) 68:947. doi: 10.1037/0022-006X.68.6.947 11142547

[32] Doering S Ohlmeier MC De Jongh A Hofmann A Bisping V. Efficacy of A trauma-focused treatment approach for dental phobia: A randomized clinical trial. Eur J Of Sci. (2013) 121:584–93. doi: 10.1111/eos.2013.121.issue-6 24206075

[33] Triscari MT Faraci P Catalisano D D’angelo V Urso V. Effectiveness of cognitive behavioral therapy integrated with systematic desensitization, cognitive behavioral therapy combined with eye movement desensitization and reprocessing therapy, and cognitive behavioral therapy combined with virtual reality exposure therapy methods in the treatment of flight anxiety: A randomized trial. . Neuropsychiatr Dis And Treat. (2015) 82:11–20. 10.2147/NDT.S93401 PMC4605250 26504391

[34] Staring A Korrelboom C Van Den Berg D Cath D Schoorl S Engelhard I. Self-esteem treatment in anxiety. Eye movement desensitization en reprocessing (Emdr) versus competitive memory training (Comet) in A randomized controlled. Cross-Over Trial. (2016) 11–20. doi: 10.1016/j.brat.2016.04.002 27155451

[35] Nazari H Momeni N Jariani M Tarrahi MJ. Comparison of eye movement desensitization and reprocessing with citalopram in treatment of obsessive–compulsive disorder. Int J Of Psychiatry In Clin Pract. (2011) 15:270–4. doi: 10.3109/13651501.2011.590210 22122001

[36] Keynejad RC Dua T Barbui C Thornicroft G. Who mental health gap action programme (Mhgap) intervention guide: A systematic review of evidence from low and middle-income countries. BMJ Ment Health. (2018) 21:30–4. doi: 10.1136/eb-2017-102750 PMC10283403 28903977

